# Interpersonal lending network dataset of a Hungarian village in a disadvantaged region based on a quantitative survey

**DOI:** 10.1016/j.dib.2023.108946

**Published:** 2023-02-02

**Authors:** Márton Gosztonyi, Dániel Havran, Zoltán Pollák, Edina Berlinger

**Affiliations:** a Asia-Europe Institute, Universiti Malaya, Jln Profesor Diraja Ungku Aziz, 50603 Kuala Lumpur, Wilayah Persekutuan Kuala Lumpur, Malaysia; b Department of Finance, Corvinus University of Budapest, Fővám tér 8, Budapest, 1093, Hungary; c Department of Finance, Budapest Business School University of Applied Sciences, Buzogány u. 10-12, Budapest 1149, Hungary

**Keywords:** Social-Network Analysis (SNA), Informal Finance, Finance of low-income households, Participatory Action Research (PAR)

## Abstract

The dataset presented in this paper consists of a network of interpersonal lending relations from a single village of a deprivated area of Hungary. The data are originated from quantitative surveys from May 2014 to June 2014. The data collection was embedded in a Participatory Action Research (PAR) which aimed to investigate the financial survival strategies of low-income households in a Hungarian village in a disadvantaged region. The directed graphs of lending and borrowing are a unique dataset that empirically captures a hidden and informal financial activity between households. The network contains 164 households and 281 credit connections among them.

**Specifications Table**

**Table:** 

Subject	Finance and Banking, Social Science, Sociology

Specific subject area	Informal lending network of households
Type of data	Table
How the data were acquired	Quantitative survey with 171 individuals aggregated to 164 households.
Data format	Raw and filtered. The responses were anonymized and aggregated to the household level.
Description of data collection	The dataset includes the directed network of informal lending relations of households in a single village in a disadvantaged region, and records socio-economic characteristics of the respondents. The interpersonal lending network data were collected based on a survey. The dataset covers approximately 2/3 of the total population of the village. The data collection was embedded in a Participatory Action Research (PAR) which aimed to investigate the financial survival strategies of low-income households.
Data source location	Settlement/Region: Kázsmárk/ Borsod-Abaúj-Zemplén County Country: Hungary
Data accessibility	Repository name: Zenodo Data identification number: 10.5281/zenodo.7550696 Direct URL to data: https://zenodo.org/record/7550696
Related research article	Berlinger E., Gosztonyi M., Havran D., Pollák Z. (2023). Interpersonal versus interbank lending networks: The role of intermediation in risk-sharing, *Emerging Markets Review,* 54, 100989, https://doi.org/10.1016/j.ememar.2022.100989

## Value of the Data

• The database [1] contains a directed graph of informal loans among households who live in the given village. The dataset combines this network with a graph of social relationships and household-level economic and social characteristics. Village-level collaboration patterns among different social groups can be analyzed using underlined structures of interpersonal borrowing and lending.
• The dataset contains informal, interest-free lending relationships between households which are usually difficult to record. As a result, the database provides insight into a transaction system that is difficult to measure.
• The dataset can be utilized for policymaking for the development of low-income households. Especially for policies promoting integrated and collaborative financial development programs that aim to focus on interdependencies. All research that aims to get a better understating of the informal finance of households can benefit from the dataset.
• Data make it possible to compare different social networks by specific economic and sociologic properties. The graph structure enables the analysis from multiple perspectives, many of which are not yet fully processed. Researchers aiming to perform a systematic review and meta-analysis study in the future can use the data well.

## 1 Objective

The database describes how households manage their liquidity shocks by providing informal loans to each other depending on their income, location, family relationship, and friendship during a given period. The database makes it possible to reveal the intermediary activities of the villagers and to analyze the topology and other graph properties of the interpersonal lending network. The database offers information on risk-sharing transactions of households with different income levels, ethnicity (Roma versus non-Roma), and location.

The dataset primarily corresponds to the study of Berlinger et al. (forthcoming) that investigates interpersonal lending activities of the households by applying methods from finance, sociology, and network science and comparing it to an interbank trading network.

## 2 Data Description

Data presented in this article include raw data in a table format prepared for further analysis. The dataset consists of the responses of the residents of Kázsmárk to a survey questionnaire, the village located in Borsod-Abaúj-Zemplén county in Hungary. The population of the settlement was around 984 in 2015, in the year of the assessment. The village is considered as cumulatively disadvantaged relative to other Hungarian rural settlements.

The livelihood of low-income families is characterized by high financial volatility, as a result, households form a strong network of informal financial relationships (credit relations) between each other. The dataset represents these socioeconomic connections as a complex system of network relations of informal financial transactions, which households use in their daily lives to cover their expenses. The partner choice logic of the network is based on economic choices made from past and present economic performances of the partners, but it is also structured by the available local socioeconomic capital of the partners. Thus, the kinship and friendship relations determine the network as well. Therefore, our dataset presents not just the informal credit transactions between the households but also the kinship and friendship connections between the households.

Households, the observation units are defined as people living at the same address. In the investigated period, researchers identified 260 addresses in the village, and all the corresponding households were approached. However, only 164 of them were willing to answer the survey. Thus, the data provide information about the lending network covering the accessible responses of the population based on voluntary reporting.

**Fig. 1:**
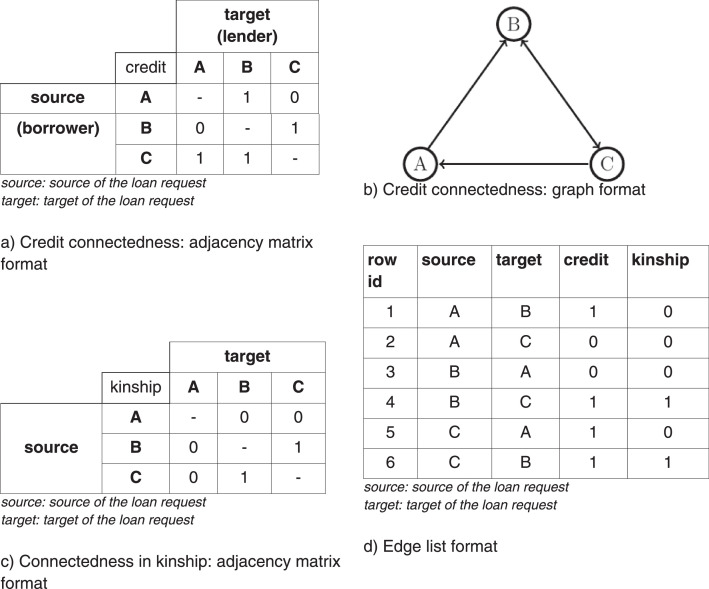
Example: logical structure of the data.

The dataset represents interactions of villagers, similar to the example shown in Fig. 1. One can describe connections of credit and kinship among A, B and C individuals in an adjacency matrix form like in parts a) and c). The same information is stored in part d) by rows. Considering the credit relationship between A and B, the first line of part a) reads as “A borrows from B”, c) reads as “A is not akin with B”, and part d) line 1 reads “A borrows from B, but A is not akin with B”.

Data containing several types of links between the households are stored in an edge list format.

The dataset consists of 13 variables detailed in Table 1. Each tie (the lending variable equals to 1) means that there is an informal, interpersonal credit transaction between the source and target households. The ties attributes contain further aspects about the households and the connections. These indicate the personal relationships between the households (kinship, friendship), the spatial distance between the households (dist, dist_p30, neighbor10, same_street), the ethnicity of the households that is based on self-assessment (target.romani, source.romanii), and the financial situation of the households (target.poor, source.poor).

Table 2 introduces the descriptive statistics. According to graph theory, the maximum number of connections (edges) in a graph of 164 nodes equals to 164*163 = 26,732. The dataset contains all the possible links (in 26,732 rows), but many of them are empty. The directed informal lending network consists of 281 edges. The kinship and friendship networks have 582 and 276 edges, respectively. Dist, dist_p30, target.poor and source.poor variables have less than 26,732 records (NAs are inserted if data is missing). The interpretation of the first line is the following: there were 281 edges among households who transacted in the observed period. The portion of the credit transactions to the total number of the possible links is 281 / 26,743 = 0.011, the mean of credit variable.

**Table 1:** Variables of the dataset.

Variable name	Variable Description	Variable type	Value Range	Value Description
source	Identifier of the borrowing household (“source of request”)	String	H1 - H260	H: household identifier, the non-respondents are omitted
target	Identifier of the lending household (“target of request”)	String	H1 - H260	H: household identifier, the non-respondents are omitted
credit	Interpersonal lending relationship between the source and the target households. “The target of request lends the source of request.”	Binary	0, 1	0: No 1: Yes
kinship	Kinship relation between the source and the target households	Binary	0, 1	0: No 1: Yes
friend	Friendship relation between the source and the target households	Binary	0, 1	0: No 1: Yes
dist	Spatial distance between the source and the target households: Euclidean distance calculated from geolocal position of the households’ location based on the addresses (latitude and longitude)	Double	0 – 0.0141, NA	numeric NA: cannot be determined
dist_p30	Spatial distance (dist. variable) is less than the 30th percentile of the distances across the dataset	Binary	0, 1, NA	0: No 1: Yes NA: cannot be determined
neighbor10	Is the target household among the 10 closest neighbours of the source household?	Binary	0, 1	0: No 1: Yes
same_street	Do both of the households live in the same street?	Binary	0, 1	0: No 1: Yes
target.romani	The target household is considered as Roma, based on self-assessment	Double	0, 0.83, 1	0: No 0.83: Mixed 1: Yes
source.romani	The source household is considered as Roma, based on self-assessment	Double	0, 0.83, 1	0: No 0.83: Mixed 1: Yes
target.poor	The target household is considered as low-income (below the poverty threshold, 70,000 HUF (∼200 EUR), based on total monthly income)	Binary	0, 1, NA	0: No 1: Yes NA: cannot be determined
source.poor	The source household is considered as low-income (below the poverty threshold, 70,000 HUF (∼200 EUR), based on total monthly income)	Binary	0, 1, NA	0: No 1: Yes NA: cannot be determined

**Table 2:** Descriptive statistics.

Statistic	N	Mean	St. Dev.	Min	Max	Sum
credit	26,732	0.011	0.102	0	1	281
kinship	26,732	0.022	0.146	0	1	582
friend	26,732	0.010	0.101	0	1	276
dist	26,406	0.006	0.004	0.000	0.014	-
dist_p30	26,406	0.299	0.458	0	1	-
neighbor10	26,732	0.061	0.240	0	1	-
same_street	26,732	0.246	0.431	0	1	-
target.romani	26,732	0.475	0.499	0.000	1.000	-
source.romani	26,732	0.475	0.499	0.000	1.000	-
target.poor	18,745	0.696	0.460	0	1	-
source.poor	18,745	0.696	0.460	0	1	-
	

If credit indicator equals to one for a “source” and a “target” household then source is called as borrower and target is as lender. Table 3 breaks down the structure of the lending network by the lending and borrowing roles of households. Altogether, 29 households borrow only, 59 menages lend only, and 70 of them intermediate (do both). More households lend (rarely), and lesser households borrow (more frequently). Thus, a borrower usually has more lenders. The directed network comprises 158 nodes, 6 households do not receive or give interpersonal loans.

**Table 3:** Number of households.

Type	Number
Borrows only	29
Lends only	59
Intermediates (borrows and lends)	70
Total of connected households (nodes)	158
Disconnected households	6
Number of responding households (actors in the dataset)	164
Number of non-responding households (not in the dataset)	96
Total households in the village	260

## 3 Experimental Design, Materials, and Methods

The data was collected in the period between 2015 May 15^th^ and June 24^th^. The data collection was embedded in a Participatory Action Research (PAR) aimed to investigate the financial survival strategies of low-income households [2], and took place in the village between June 2014 and October 2015. The data includes 164 households covering the 2/3 of the local population, The data collection followed the scientific methodology, hence it is representative for the whole village. For a deeper understanding of data design, we briefly discuss the PAR methodology and our PAR research in the following.

PAR research methodology creates a process based on cooperative learning which combines different knowledge types and experiences [3,4]. Furthermore, PAR breaks the classical dichotomy of the ‘researcher’ and the ‘researched’ and attempts to resolve the willingly or unwillingly created hierarchical relationship between them [[5], [6], [7]]. PAR is a continuous reflexive process, in which researchers from outside the community and researchers from the community work together to explore and analyze local problems with scientific methodologies and implement actions to make a change [8]. Thus, the characteristic of PAR is that it adapts traditional scientific research methods innovatively to local circumstances to guide solving local problems [6,9]. During the process, a cooperation is developed, where the aim of the research project, and the collection and use of data are all determined via communal decisions. Therefore, PAR can: 1) generate sophisticated and high-quality data through qualitative and quantitative methodology, even in segments that belong to hidden structures of the social fabric, and: 2) lead to the initiation or facilitation of community-based developments or advocacy processes that implement positive social changes [10,11].

Our PAR process was conducted to achieve positive social changes locally in which 1) the participants consciously used the methodology of social scientific research – in this sense, it was a structured societal research project, and 2) the process was based on cooperation and mutual learning of the locals and outsider researchers to create positive change.

The PAR began in late June 2014 by organizing a “meta-research group” that got together weekly in Budapest, Hungary to prepare the research project. The meta-research group provided a platform to recruit volunteer experts for later research and it also created a research methodology and theoretical framework for the project. In July 2014, we sat down several times with local key figures of the village to talk about the PAR process. Consequently, the work of the meta-research group could gradually transform into the actual PAR group in the village in September. We held our PAR meetings every Friday in the village. We held 39 group meetings until late October 2015, in the course of which we covered a full PAR process from group formation to local problem analysis by formulating research questions, hypotheses, and research methodologies, and by preparing the tools used for data collection, later analyzing them and presenting our result on conferences, and finally launching a local economic development program based on our scientific results.

The PAR researchers from the village represented the Roma families living in the village. Local PAR researchers did not have any previous research experience, the average formal education within the group was the completed primary school education or a vocational school. Most group members came from low-income households. Our PAR process followed a rather precisely applied structure that we can divide into nine major phases (Table 4).

**Table 4:** Major phases of the PAR process.

Phase	Description
1^st^ Phase	Group formation
2^nd^ Phase	Definition of the research topic and research questions
3^rd^ Phase	Action planning
4^th^ Phase	Theory and methodology
5^th^ Phase	Development of the measurement tools
6^th^ Phase	Retreat at Budapest
7^th^ Phase	Data collection
8^th^ Phase	Data analysis, presentations at conferences
9^th^ Phase	Action: Implementing social enterprises

The research topic was outlined early on in the process, as the PAR group wanted to focus on local livelihood and employment opportunities. After elaborating on the research topic, the following meetings revolved around collecting the formal and informal, seasonal and permanent employment opportunities, and we gradually mapped the formal and informal savings, loans and credit opportunities available locally. By the end of this process, we modelled the complex system of local financial survival strategies. In the third phase (planning the action) we decided to develop local projects that broaden the livelihood opportunities of residents through increasing employment opportunities. Thus, we developed five social business ideas. In the fourth phase, we studied scientific theories and methodologies so that we could determine the appropriate methodology for our research question and hypothesis. In the fifth phase, the PAR group developed its research tools. We agreed on a two-wave data collection process for our research. In the first data collection wave, we used financial diaries and in the second wave, we used the survey method. Both measurement tools were created by the PAR group. The financial diaries were queried on a sample of the local population while the network survey data was collected among the total population. In the sixth phase, we implemented a two-day retreat in Budapest which involved evaluating the group meetings, as well as discussing, planning, and scheduling upcoming tasks and fine-tuning our questionnaire. We also could visit projects that served as inspirations for our social enterprise, and we also presented our social enterprise projects to potential donors. In the seventh phase of the PAR process, we conducted the data collection. In the eighth phase, we analyzed our data and presented our results at scientific conferences. Thanks to our intensive lobbying, we received financial support for our projects and could start the social enterprises at phase nine. This part started in October 2015 and ran for more than two years.

In summary, our PAR research generated a long-term social development process, based on our research results, that could broaden the livelihood opportunities of residents and increase employment opportunities at the local level.

The development of the questionnaire used for network data collection was thus embedded in a long PAR process. The financial difficulties of families living in the village are reflected in the data we measured: based on their formal income, 83% of the families live under the 2015 Hungarian poverty threshold per person. 57% of the families had a low level of education (primary education or less than primary education). Among the families, there was a high proportion of those living in difficult working possibilities (the number of unemployed was 41% in the sample). The average age of the respondents was 45.5 years (SD: +- 15,2 years) [2]. However, because of reasons of confidentiality, we make only part of the questionnaire data publicly available. The presented part of the dataset was constructed by the aggregation of the following questionnaire questions:

• (Q1) To whom do you lend a smaller amount (cash, transportation, food)?
• (Q2) From whom do you borrow a smaller amount (cash, transportation, food)?
• (Q3) From whom do you borrow a larger amount?

**Fig. 2:**
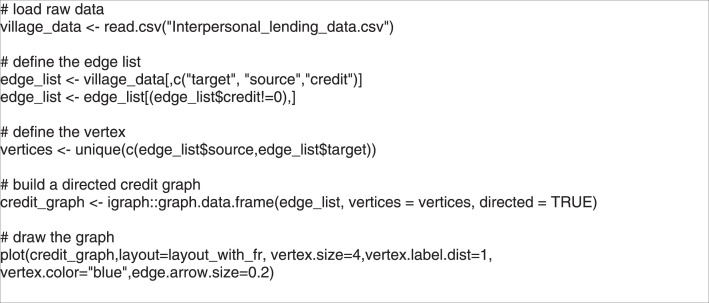
Code of loading the data into R as an igraph object and plot the graph of lending.

The responses were recorded using the name-generation method [12]. The recorded responses for questions Q1, Q2 and Q3 were placed in a container variable called “credit” with two values (1=there is a credit relation, 0=there is not a credit relation). We aggregated the personal data to household level data in the R software environment. The dataset was stored in a comma separated values (CSV) text file format. Fig. 2. shows an example code on loading the data and using it as a network object in R.

It follows from all this that our data gives a locally representative sample about the most disadvantaged social strata in Hungary, which is 30-35% of the entire Hungarian society [13]. Marginality, day-to-day survival and solving permanent financial crisis situations have become a daily experience for these households. Furthermore, these families live in a multi-segment cumulatively disadvantaged situation, as they are simultaneously affected by the scarcity of access to jobs, housing, health services, education, and formal financial institutions [13]. As a result, they are limited in their access to basic goods and services [14].

## Ethics Statements

All survey participants were thoroughly informed about the content and the scope of the study before participation. Thus, informed consent was obtained from the participants prior to the surveys. Participation was completely voluntary. Moreover, the anonymity of the data is guaranteed by excluding all personally identifiable information of respondents. Therefore, no personal, sensitive information is included in the dataset. The ethical committee of the research was the Doctoral School of Sociology of Corvinus University of Budapest (protocol number: DSS2015/003).

## CRediT Author Statement

**Márton Gosztonyi**: Conceptualization, Methodology, PAR research, Data gathering (survey management), Data curation, Data Curation, Software, Validation Writing - Review & Editing; **Dániel Havran:** Methodology, Data curation, Software, Validation, Data Curation, Writing - Review & Editing; **Zoltán Pollák:** Methodology, Data curation, Software, Validation, Data Curation, Writing - Review & Editing, Visualization; **Edina Berlinger:** Conceptualization, Methodology, Validation, Writing - Review & Editing, Supervision.

## Declaration of Competing Interest

The authors declare that they have no known competing financial interests or personal relationships that could have appeared to influence the work reported in this paper.

## Data Availability

Interpersonal lending network (Original data) (Zenodo).

## Appendix Supplementary Materials

Image, application 1
