# Supplementary material for: Interpersonal lending network dataset of a Hungarian village in a disadvantaged region based on a quantitative survey
Source: Data Brief. 2023 Feb 2;47:108946. doi: 10.1016/j.dib.2023.108946 (PMC9945707; doi:10.1016/j.dib.2023.108946)
Supplement: Supplementary file 1 [file mmc1.docx]

*Interviewer: fill it out before the interview starts!*

| A.1Name of the interviewer: |  |
| --- | --- |
| A.2Name of the interviewee: |  |
| A.3 Date of questioning: | 2015. |
| A.4 The place of residence of the interviewee: (= street, house number!) |  |

**BLOCK A: Network Questionnaire**

***Work***

- **Q1**: If you're looking for any job (permanent or casual), who do you ask?
- **Q2**: If there is a job that 3 people would have to do, who would be the 2 people who would choose beside you?

*Questioner: enter the name on sheet "A*" *and mark the columns "K1" and K2".*

***Advice, information, help***

- **Q3**: From whom do you find out the most important news in the village?
- **Q4**: Who do you trust if you have been harmed or when you face injustice?

*Questioner: enter the name on sheet "A*" *and mark the columns "K3" and K4".*

***Help with the house***

- **Q5**: Who have helped you to renovate or build your house on a back-and-forth basis?

*Questioner: enter the name on sheet "A*" *and mark the columns "K5".*

***Help around the house***

- **Q6**: Who do you ask to help you with work around the house, or take care of the kids while you're away?

*Questioner: enter the name on sheet "A*" *and mark the columns "K6".*

***Neighboring***

- **Q7**: Many people in the village often visit each other.
  Who do you go to? Who do you usually see, who do you jump into?

*Questioner: enter the name on sheet "A*" *and mark the columns "K7".*

***Gift, loan***

- **Q8**: Who do you usually transfer your brew to?
- **Q9**: To whom *do* you lend 1,000 - 2,000 forints? (freight, food)
- **Q10**: Whom do you *get* a small loan from if you get squeezed? (money, freight, food)
- **Q11**: Whom can you borrow a larger amount of money if you need it?

*Questioner: enter the name on sheet "A*" *and mark the columns "K8","K9", "K10", "K11" .*

***Sadness***

- **Q12**: Whom do you turn to if you're sad, broken, and want to talk to someone about it?

*Questioner: enter the name on sheet "A*" *and mark the columns "K12".*

***K13***

- **Q13**: Is there anyone else who is important to you and is not on the list?

If so, could you add it to the list?

What are some of the things that you usually do together with that person or that you share with her/him?

*Questioner: enter the name on sheet "A*" *and mark the columns "K13".*

**BLOCK A: Background variables**

The following questions relate to the people you mentioned.

1. **What is your relationship to these persons?**

| 1) Spouse  | 4) Brother  | 6) Friend  |
| --- | --- | --- |
| 2) Parent | 5) Other family member  | 7) Neighbor  |
| 3) Child |  | 8) Familiar  |

*About: mark the relationship in the "* ***relationship*** *" column of*  tab A.

*"Ego" can be related to one person in several ways, so you can nominate more than one (eg.*  *neighbor and friend at the same time)*

1. **Where does he/she live in Kázsmárk?**

*About: enter the street and house number in the* "***Street***,  ***hsz, settlement****" column on sheet "A". If you don't live in Kázsmárk, enter the settlement!*

1. **How frequent your contacts with the people listed?** Mark 5 for those with whom you have a very frequent, strong relationship and 1 for those with whom you have a weak relationship.

*About: circle the number in the* "***Frequency****" column of* sheet A *n!*

**Tab A to record Network NAME GENERATOR**

| ***Persons*** | **K1** | **K2** | **K3** | **K4** | **Q5** | **K6** | **K7** | **K8** | **K9** | **K10** | **K11** | **K12** | **K13** | **relationship** | **Street, settlement** | **Frequency** |
| --- | --- | --- | --- | --- | --- | --- | --- | --- | --- | --- | --- | --- | --- | --- | --- | --- |
| 1. ............................................. |  |  |  |  |  |  |  |  |  |  |  |  |  | 12345678 |  | 12345 |
| 2. ............................................. |  |  |  |  |  |  |  |  |  |  |  |  |  | 12345678 |  | 12345 |
| 3. ............................................. |  |  |  |  |  |  |  |  |  |  |  |  |  | 12345678 |  | 12345 |
| 4. ............................................. |  |  |  |  |  |  |  |  |  |  |  |  |  | 12345678 |  | 12345 |
| 5. ............................................. |  |  |  |  |  |  |  |  |  |  |  |  |  | 12345678 |  | 12345 |
| 6. ............................................. |  |  |  |  |  |  |  |  |  |  |  |  |  | 12345678 |  | 12345 |
| 7. ............................................. |  |  |  |  |  |  |  |  |  |  |  |  |  | 12345678 |  | 12345 |
| 8. ............................................. |  |  |  |  |  |  |  |  |  |  |  |  |  | 12345678 |  | 12345 |
| 9. ............................................. |  |  |  |  |  |  |  |  |  |  |  |  |  | 12345678 |  | 12345 |
| 10. ............................................. |  |  |  |  |  |  |  |  |  |  |  |  |  | 12345678 |  | 12345 |
| 11. ............................................. |  |  |  |  |  |  |  |  |  |  |  |  |  | 12345678 |  | 12345 |
| 12. ............................................. |  |  |  |  |  |  |  |  |  |  |  |  |  | 12345678 |  | 12345 |
| 13. ............................................. |  |  |  |  |  |  |  |  |  |  |  |  |  | 12345678 |  | 12345 |
| 14. ............................................. |  |  |  |  |  |  |  |  |  |  |  |  |  | 12345678 |  | 12345 |
| 15. ............................................. |  |  |  |  |  |  |  |  |  |  |  |  |  | 12345678 |  | 12345 |
| 16. ............................................. |  |  |  |  |  |  |  |  |  |  |  |  |  | 12345678 |  | 12345 |
| 17. ............................................. |  |  |  |  |  |  |  |  |  |  |  |  |  | 12345678 |  | 12345 |
| 18. ............................................. |  |  |  |  |  |  |  |  |  |  |  |  |  | 12345678 |  | 12345 |
| 19. ............................................. |  |  |  |  |  |  |  |  |  |  |  |  |  | 12345678 |  | 12345 |
| 20. ............................................. |  |  |  |  |  |  |  |  |  |  |  |  |  | 12345678 |  | 12345 |

1. **Now I would like to ask about the employment and incomes of people living in your household**

*Q:a person can have constant, casual and self-employed activity at the same time!*   *Enter thez amounts, in HUF!*

| 1.1  Code | 1.2.  What is his/her name? | 1.3.  Does he/she has **a permanent** job? (Pension included !) | 1.4.  If so, how much does she/he receive per month? | 1.5. Does he/she receive any state or local government **support**? | 1.6.  What is the total amount of these gov. support? | 1.7.  Does he/she used to  or **doing casual** work? | 1.8. If so, how much does he/she usually receive in a month? | 1.9.  **Self-occupied**? | 1.10.  If so, how much does he/she usually receive in a month? |
| --- | --- | --- | --- | --- | --- | --- | --- | --- | --- |
| **01** |  | Y N |  | Y N |  | Y N |  | Y N |  |
| **02** |  | Y N |  | Y N |  | Y N |  | Y N |  |
| **03** |  | Y N |  | Y N |  | Y N |  | Y N |  |
| **04** |  | Y N |  | Y N |  | Y N |  | Y N |  |
| **05** |  | Y N |  | Y N |  | Y N |  | Y N |  |
| **06** |  | Y N |  | Y N |  | Y N |  | Y N |  |
| **07** |  | Y N |  | Y N |  | Y N |  | Y N |  |
| **08** |  | Y N |  | Y N |  | Y N |  | Y N |  |

2. So, overall, if you count everything together, how much does your household live on in a month? ......................................... HUF

3. In the last 12 months, has the household grown any plants in the kitchen garden, in a small garden in
order to sell it or to supplement its consumption at home? 1. Yes 2. No

4. Do you keep (farm) animals? 1. Yes 2. No

5. You are in the village:

1. Feel more like rich 
2. feels like belonging to the middle class 
3. or feel poor 

6. How many friends do you have? .............................................

**7. How often do you use the following services?**  *(in the last 1 year)*

|  | **Weekly or more frequent** | **Every 2 weeks** | **Monthly** | **Every 1/4 year** | **Every six months** | **Annually** | **Less often (enter!** **)** | **Never/None** |
| --- | --- | --- | --- | --- | --- | --- | --- | --- |
| 7.1. How often do you use your **bank account**? | 1 | 2 | 3 | 4 | 5 | 6 |  | 9 |
| 7.2. How often do you pay **for insurance**? | 1 | 2 | 3 | 4 | 5 | 6 |  | 9 |
| 7.3. How often **do you set aside** at home  (e.g. for wood, home renovation, holidays)? | 1 | 2 | 3 | 4 | 5 | 6 |  | 9 |
| 7.4. How often do you get **a quick loan**? | 1 | 2 | 3 | 4 | 5 | 6 |  | 9 |
| 7.5. How often do you use your **credit card**? | 1 | 2 | 3 | 4 | 5 | 6 |  | 9 |
| 7.6. How often do you **buy on credit** in Metro, Auchan, Mivas? | 1 | 2 | 3 | 4 | 5 | 6 |  | 9 |
| 7.7. If you urgently need money, how often do you use the **pawnshop**? | 1 | 2 | 3 | 4 | 5 | 6 |  | 9 |
| 7.8. How often **do you ask someone** to  **buy something for you, for cash**? | 1 | 2 | 3 | 4 | 5 | 6 |  | 9 |
| 7.9. When you have nowhere to reach anymore, how often do you ask for **money with interest**? | 1 | 2 | 3 | 4 | 5 | 6 |  | 9 |
| 7.10. How often do you ask **your boss for a down payment**? | 1 | 2 | 3 | 4 | 5 | 6 |  | 9 |
| 7.11. How often do you ask the local mayor for a loan? | 1 | 2 | 3 | 4 | 5 | 6 |  | 9 |
| 7.12. How often do you shop in the **store** without paying right away, but then **taking** in the price later? | 1 | 2 | 3 | 4 | 5 | 6 |  | 9 |
| 7.13. How often do you usually **lend** a small amount **to a neighbor or friend**? | 1 | 2 | 3 | 4 | 5 | 6 |  | 9 |
| 7.14. How often **do you get** a small amount of money from **a neighbor/friend**? | 1 | 2 | 3 | 4 | 5 | 6 |  | 9 |
| 7.15. How often do you ask someone to  **buy** for you, **on credit**? | 1 | 2 | 3 | 4 | 5 | 6 |  | 9 |
| 7.16. How often **do you share** **your salary** /**money** with others so that someone? | 1 | 2 | 3 | 4 | 5 | 6 |  | 9 |
| 7.17. How often do you **buy** any goods **from a house** ? | 1 | 2 | 3 | 4 | 5 | 6 |  | 9 |
| 7.18. How often **do you buy** something to **sell** to someone else? | 1 | 2 | 3 | 4 | 5 | 6 |  | 9 |

**8. Household - Please list your household members by their age of birth.**

* Member of the **household** = **(1st)** who lives under a "roof"; and  **(2nd)** gets involved in food costs and consumption; and **(3)** contribute to the family resources.

| 8.1.  Code | 8.2.  List all the people who fit the above 3 criteria | 8.3.  What is his/her relationship with the head of the family | 8.4.  Sex | 8.5.  Which  Year he/she was  born? | 8.6.  What is a  family  Status? | 8.7.  Does she/he have children? | 8.8.  Employment status | 8.9. Highest completed education level | 8.10.  In which settlement did he live after his birth? | 8.11.  Religion |
| --- | --- | --- | --- | --- | --- | --- | --- | --- | --- | --- |
| ***Code*** | ***NAME*** | ***Write Codes!*** |  | ***Y y y y*** | ***Write Codes!*** | ***Number*** | ***Write Codes!*** | ***Write Codes!*** | ***Enter the name of the settlement*** | ***Write Codes!*** |
| **01** |  |  | F/N |  |  |  |  |  |  |  |
| **02** |  |  | F/N |  |  |  |  |  |  |  |
| **03** |  |  | F/N |  |  |  |  |  |  |  |
| **04** |  |  | F/N |  |  |  |  |  |  |  |
| **05** |  |  | F/N |  |  |  |  |  |  |  |
| **06** |  |  | F/N |  |  |  |  |  |  |  |
| **07** |  |  | F/N |  |  |  |  |  |  |  |
| **08** |  |  | F/N |  |  |  |  |  |  |  |
| **09** |  |  | F/N |  |  |  |  |  |  |  |
| **10** |  |  | F/N |  |  |  |  |  |  |  |
|  |  | **8.3.** **Codes** |  |  | **8.6.** **Codes** | **8.8.** **Codes** | | **8.9.** **Codes** | | **8.11.** **Codes** |
|  |  | 01 = Head of family  02 = Married-  partner  03 = Children  05 =Grandchild  Other: |  |  | 01 = Never  married  02 = Partner connection  03 = Widow  Other: | 01 = Unemployed/looking for a job  02 = Full job  03 = Public worker  04 = part-time job | 05 = Self-employed  06 = Student  07 = Retired  Other: | 01 = less than prim. school  02 = prim. school  03 = Apprenticeship | 04 = Gimnasium  05 = College,  university  07 = OKJ. | 01 = Roman chat.  02 = Reformed  03 = Greek-c.  04 = Evan-  Gelic  05 = Not  religious  Other: |

9. How often do you find yourself in need of a loan to pay off your daily expenses and to live on a daily basis?

1. Every week 
2. Every month 
3. Every 2-3 months 
4. Once or twice a year 
5. Less often 
6. Not occur 

9.1. Do you have bank loan in your household?

1. Yes
2. No

9.2. Please list what bank loans do you have?

1. ..............................................................................
2. ..............................................................................
3. ..............................................................................
4. ..............................................................................
5. ..............................................................................

10. When was your house built?

1. Before 1945 
2. 1945 to 1990 
3. After 1990, and that:...................... *(Approx: Enter the year)*

11. How many rooms is your house?.................................. *(Number of rooms)*

12. Do you feel that you belong to *one or more* nationalities/ethnicity among the following?

1. Hungarian 
2. Roma / Gypsy 
3. Ukrainian 
4. Slovakian 
5. Other, namely:...............................
6. I prefer not to answer

**Thank you for your answers, and thank you,**

**That you helped the work of our research group!**

**If you have any questions about our research,**

**feel free to ask the members of the research team,**

**or come to one of our Friday group meetings !**
